# Key Population Size Estimation to Guide HIV Epidemic Responses in Nigeria: Bayesian Analysis of 3-Source Capture-Recapture Data

**DOI:** 10.2196/34555

**Published:** 2022-10-26

**Authors:** Anne F McIntyre, Andrew Mitchell, Kristen A Stafford, Samuel Uchenna Nwafor, Julia Lo, Victor Sebastian, Amee Schwitters, Mahesh Swaminathan, Ibrahim Dalhatu, Man Charurat

**Affiliations:** 1 Division of Global HIV and TB Center for Global Health Centers for Disease Control and Prevention Atlanta, GA United States; 2 Institute of Human Virology University of Maryland School of Medicine Baltimore, MD United States; 3 Center for International Health, Education, and Biosecurity University of Maryland School of Medicine Baltimore, MD United States; 4 Department of Epidemiology and Public Health University of Maryland School of Medicine Baltimore, MD United States; 5 Center for International Health, Education, and Biosecurity University of Maryland School of Medicine Abuja Nigeria; 6 Division of Global HIV and TB Center for Global Health Centers for Disease Control and Prevention Abuja Nigeria

**Keywords:** sex workers, men who have sex with men, people who inject drugs, HIV, population size, population, data, female, men, drugs, drug injection, epidemic, Nigeria

## Abstract

**Background:**

Nigeria has the fourth largest burden of HIV globally. Key populations, including female sex workers, men who have sex with men, and people who inject drugs, are more vulnerable to HIV than the general population due to stigmatized and criminalized behaviors. Reliable key population size estimates are needed to guide HIV epidemic response efforts.

**Objective:**

The objective of our study was to use empirical methods for sampling and analysis to improve the quality of population size estimates of female sex workers, men who have sex with men, and people who inject drugs in 7 states (Akwa Ibom, Benue, Cross River, Lagos, Nasarawa, Rivers, and the Federal Capital Territory) of Nigeria for program planning and to demonstrate improved statistical estimation methods.

**Methods:**

From October to December 2018, we used 3-source capture-recapture to produce population size estimates in 7 states in Nigeria. Hotspots were mapped before 3-source capture-recapture started. We sampled female sex workers, men who have sex with men, and people who inject drugs during 3 independent captures about one week apart. During hotspot encounters, key population members were offered inexpensive, memorable objects unique to each capture round. In subsequent rounds, key population members were offered an object and asked to identify objects received during previous rounds (if any). Correct responses were tallied and recorded on tablets. Data were aggregated by key population and state for analysis. Median population size estimates were derived using Bayesian nonparametric latent-class models with 80% highest density intervals.

**Results:**

Overall, we sampled approximately 310,000 persons at 9015 hotspots during 3 independent captures. Population size estimates for female sex workers ranged from 14,500 to 64,300; population size estimates for men who have sex with men ranged from 3200 to 41,400; and population size estimates for people who inject drugs ranged from 3400 to 30,400.

**Conclusions:**

This was the first implementation of these 3-source capture-recapture methods in Nigeria. Our population size estimates were larger than previously documented for each key population in all states. The Bayesian models account for factors, such as social visibility, that influence heterogeneous capture probabilities, resulting in more reliable population size estimates. The larger population size estimates suggest a need for programmatic scale-up to reach these populations, which are at highest risk for HIV.

## Introduction

Key populations, including female sex workers, men who have sex with men, and people who inject drugs, are disproportionately vulnerable to HIV infection compared to the general population due to stigma, discrimination, and criminalization of key population–defining behaviors [1-5]. The poor social visibility and high mobility of these populations obscure understanding of the magnitude and distribution of their HIV burden. To guide focused and appropriately scaled HIV epidemic response efforts for these key populations, program and policy development requires reliable, empirical population size estimates [6-8].

Nigeria has the fourth-largest burden of HIV globally, with an estimated 1.9 million people living with HIV [9]. It is a mixed [10] epidemic, with a relatively low HIV prevalence of 1.4% among the adult general population [11] but higher estimated prevalence among key populations: 15.5% among female sex workers, 25% among men who have sex with men, and 10.9% among people who inject drugs [12]. The Government of the Federal Republic of Nigeria (GoN)’s National HIV and AIDS Strategic Framework 2017-2021 [13] outlines the plan to “fast-track the national response towards ending AIDS in Nigeria by 2030” and includes focused interventions to increase testing and treatment for key populations. Results of the 2018 Nigeria HIV/AIDS Indicator and Impact Survey (NAIIS) demonstrated a lower HIV prevalence in the general population than previously reported [11,14], suggesting that key populations are an important contributor to the epidemic. As a result, focus is shifting to key populations as an opportunity to have the greatest impact on HIV epidemic control. With that shift comes the need to have better data to inform HIV programming, including reliable population size estimates.

Previous efforts to estimate key population sizes in Nigeria [15-17] were largely based on programmatic mapping [18] with enumeration of female sex workers and people who inject drugs at specific venues. These approaches did not provide uncertainty bounds and likely resulted in underestimates, as only the members of each population who could be identified at specific venues were counted. During the 2015 effort, traditional (2-source) capture-recapture was used to estimate the population size of men who have sex with men in 8 states of Nigeria [15]. In 2009, 2-source capture-recapture was used to estimate the population size of men who have sex with men in 3 large Nigerian cities [19]. Since these efforts, improved methods like 3-source capture-recapture (3S-CRC) sampling have been used to estimate population sizes of female sex workers in sub-Saharan African countries, such as South Sudan [20], Uganda [21], and Rwanda [22]. Compared to programmatic mapping and enumeration, which incorporate only those populations present and visible at a venue, the 3S-CRC sampling approach is an improvement, because it accounts for lack of social visibility, thereby producing more realistic population size estimates to inform HIV prevention and treatment programs for key populations. These examples of successful population size estimates using 3S-CRC data were analyzed using Bayesian nonparametric latent-class models [23]. We expanded use of 3S-CRC sampling of key populations from female sex workers in East Africa [20-22] to female sex workers, men who have sex with men, and people who inject drugs in Nigeria.

The methods for sampling and analysis we present here have the potential for far broader use than just key population size estimation of those at high risk of HIV in Nigeria or other countries. These methods may be applied to estimate the prevalence or incidence of HIV diagnoses in specific age groups, such as children [24]; people with diabetes [25], ophthalmologic conditions [26], or spinal cord injuries [27]; people experiencing homelessness [28]; people exposed to environmental hazards, such as lead, in homes [29]; and people who have died from injuries [30]. The methods can also be used to determine the completeness of disease reporting, such as for tuberculosis notification [31].

The objective of our study was to use empirical methods for sampling and analysis to improve the quality of population size estimates of female sex workers, men who have sex with men, and people who inject drugs in 7 states (Akwa Ibom, Benue, Cross River, Lagos, Nasarawa, Rivers, and the Federal Capital Territory [FCT]) in Nigeria for program planning and to demonstrate improved statistical estimation methods. These 7 states represent the US President’s Emergency Plan for AIDS Relief (PEPFAR) priority states in Nigeria based on unmet needs for HIV services in areas with high HIV prevalence.

## Methods

### Study Design

We sampled female sex workers, men who have sex with men, and people who inject drugs using 3S-CRC in the 7 states from October through December 2018. Traditional (ie, 2-source) capture-recapture methods for human population size estimates, where the term “capture” refers to the confirmed counting of a specific person, are described well elsewhere [19,32-35]. Accurate estimates generated from this method are challenged by violations of capture-recapture assumptions: that the study population is closed, the captures are independent, the probability of capture is similar across the entire population (ie, captured and not captured) for each source, and capture histories are accurate. The addition of one or more data sources strengthens the method, relaxing the independence assumption, as interaction can be accounted for in the statistical models.

A formative assessment that included focus group discussions and key informant interviews was used to engage key population communities in planning the implementation of this study, and was also used to identify acceptable unique objects for capture-recapture. Comprehensive measures were implemented to ensure the safety and security of the study teams and participants who were members of groups whose behavior is highly stigmatized and criminalized. The unique objects were distributed in hotspots, defined as venues where key population members congregate or engage in sexual or injecting behaviors. With the most recent lists (from 2013 and 2015) produced by efforts to map key population hotspots [15,16] as a foundation, we engaged 261 key population members from 36 key population–led, community-based organizations in the 7 states for a comprehensive review and update of hotspot information during August 2018 [36]. Local government areas were grouped into 3 zones per state to manage staff, equipment, and travel for the mapping activity, which facilitated better control over missing or duplicate hotspot visits. The mapping teams consisted of 2 to 3 key population community members who used updated lists and tablets with the REDCap survey tool (Research Electronic Data Capture; Vanderbilt University); they were assigned to visit several hotspots per day depending on their proximity and travel time. The mapping teams visited every local government area in every zone of every state and documented geographic coordinates, name (formal or informal), address, peak days and times of key population activity, and an estimate of the number of key population members present during the visit. There was at least one hotspot identified in every local government area in each of the 7 states; all hotspots were visited and documented during this activity. Most local government areas were a combination of urban and rural areas, although boundaries between the two were often difficult to distinguish, hindering our efforts to define each hotspot as urban or rural. Information collected during mapping was reconciled, deduplicated, and verified. The updated list of hotspots informed venue sampling, allocation of the 261 trained key population data collectors, and scheduling of hotspot visits to distribute the unique objects for each of the 3 sampling rounds of the 3S-CRC activity.

All hotspots identified during the mapping and validation exercise that were still active at the time of 3S-CRC were included in venue sampling for each capture round. During all sampling rounds, if a hotspot was inactive when the team arrived, this was documented, and the team moved to the next hotspot on the schedule. If new hotspots were discovered during any sampling round, the list was updated, but the hotspot was not sampled for 3S-CRC.

### Study Subjects

We sampled key population members from hotspots based on the mapping and validation activity completed immediately prior to 3S-CRC sampling [36].

Female sex workers were defined as any woman (female sex at birth) aged 18 years or older who received money or goods in exchange for sexual services, either regularly or occasionally, in the 12 months preceding this activity. All 15- to 17-year-old girls who reported receiving money or goods in exchange for sexual services were defined as sexually exploited minors (referrals to support services were provided) and were counted as sex workers for the purposes of population size estimates. Men who have sex with men were defined as any man (male sex at birth) aged 15 years or older who had engaged in oral or anal (receptive or insertive) sex, or both, with another man at least once in the previous 12 months. People who inject drugs were defined as any person aged 15 years or older who injected drugs (ie, illicit, nonprescribed, or illegal substances) at least once in the preceding 12 months.

Individuals were excluded if they reported having already been captured during a given round; if they failed to meet their key population definition, did not consent to participate, or were aged less than 15 years; if the hotspot they had been captured in during a previous round was no longer active; or if the key population team was unable to return in subsequent rounds.

### Sampling

To estimate the sample size for each capture round, we compared the recorded number of key population members present in each hotspot during mapping and validation with previous population size estimates [15,16] to produce reasonable approximations for each state and key population.

During the 3 capture rounds, we aimed to have 3 people per distribution team, with at least two being members of the same key population at the assigned hotspot to optimize acceptance of the unique objects. During encounters with key population peers in hotspots, team members described the population size estimate activity and asked the peers whether they had been approached during this sampling round. If not, the team members obtained verbal consent from those who agreed to participate and administered a brief survey to capture age, sex at birth, gender identity, education, primary source of income, local government area and state of residence, whether the individual traveled to the current or another local government area for work, and whether they currently injected drugs or engaged in sex work. Participants were offered an inexpensive and memorable object (a “gift”) unique to each of the 3 capture rounds and 3 key populations (ie, female sex workers, men who have sex with men, and people who inject drugs). This process was repeated by different unique-object distribution teams for each of 3 distinct capture rounds, performed approximately one week apart.

In subsequent rounds, participants were asked to show or describe the objects they had received during all previous rounds; affirmative responses were tallied upon correct identification of the object for each previous round.

### Measures

A capture was defined as acceptance of the gift offered by the data collection team. Recaptures (ie, second and third captures) were defined as either showing distributors the gifts from previous capture rounds or correctly describing and identifying the gift from a set of 20 pictures of various unique objects (some with the same object in a different color and some with completely different objects).

#### Data Collection

Distributors recorded participant responses on tablets with REDCap software [37,38] and uploaded the data to a secure central server after each encounter. All encounters were documented with consent.

#### Data Analysis

Individual encounter data were exported to SAS (version 9.4; SAS Institute Inc). After reviewing the data for missing or nonsensical responses, individual encounters were subdivided by state, key population group (ie, female sex workers, men who have sex with men, and people who inject drugs), age group (15-24 years and ≥25 years), and, for people who inject drugs, sex. Aggregated data sets detailing counts of each capture and recapture combination were produced for each subset of data. For the 3 capture rounds, matrices with 4 columns (round 1, round 2, round 3, and total count) and 7 rows representing each potential combination of captures (with 1 representing “captured” and 0 “not captured”) were produced. Aggregated data sets summarizing data counts in each capture round combination were produced for each subset.

Bayesian nonparametric latent-class models [23], which are able to account for capture heterogeneity, were used to produce key population size estimates from aggregated 3S-CRC data. State-specific models were generated for each key population and disaggregated by age group. The results presented here represent combined estimates for male and female people who inject drugs, as there were insufficient sample sizes for female people who inject drugs. The posterior distribution for latent-class models may produce wide 95% credible intervals with a long tail, so we calculated 80% highest density intervals (HDIs) to facilitate interpretation of population size estimates and improve ease of application to HIV programming.

All statistical analyses of aggregated data sets to generate median population size estimates with 80% HDIs were performed with R statistical software (version 3.4.4; R Foundation for Statistical Computing) using packages for latent-class models for capture-recapture [39] and HDI (HDInterval) [40].

### Ethical Considerations

This study was approved by the National Health Research Ethics Committee Nigeria and the Institutional Review Board of the University of Maryland at Baltimore (HP00080293). The study was reviewed in accordance with the US Centers for Disease Control and Prevention (CDC) human research protection procedures and was determined to be research, but CDC investigators did not interact with the human subjects or have access to identifiable data for research purposes.

## Results

### Enrollment

Overall, 9015 hotspots identified during mapping were sampled and included in the analysis, including 5946 (66%) hotspots for female sex workers, 1256 (13.9%) hotspots for men who have sex with men, and 1813 (20.1%) hotspots for people who inject drugs. A total of 310,140 individual encounters from capture rounds 1, 2, and 3 were included in the analyses (Table 1). There were 88,805 individuals excluded from the analysis due to nonconsent, ineligibility, or captures and recaptures from hotspots that were not visited in all 3 rounds due to accessibility, security, or time constraints.

**Table 1:** Numbers of encounters and hotspots by state and key population.

State	FSWs^a^, n	FSW hotspots, n	MSM^b^, n	MSM hotspots, n	PWID^c^, n	PWID hotspots, n	Total subjects, n	Total hotspots, n
Akwa Ibom	32,635	690	11,760	230	14,659	307	59,054	1227
Benue	35,284	845	9726	221	14,059	272	59,069	1338
Cross River	13,344	544	3670	195	10,142	291	27,156	1030
Federal Capital Territory	25,800	837	3427	100	3076	85	32,303	1022
Lagos	36,147	1171	2444	83	7363	149	45,954	1403
Nasarawa	25,609	929	4600	232	9790	283	39,999	1444
Rivers	30,447	930	7733	195	8425	426	46,605	1551
Total	199,266	5946	43,360	1256	67,514	1813	310,140	9015

^a^FSW: female sex worker.

^b^MSM: men who have sex with men.

^c^PWID: people who inject drugs.

### Population Size Estimates

Modeled median population size estimates with 80% HDIs for each state are presented for female sex workers (Table 2), men who have sex with men (Table 3), and people who inject drugs (Table 4). The tables include general population census projections from 2018 for the relevant sex or sexes (ie, men only for men who have sex with men, women only for female sex workers, and both for people who inject drugs) and age groups (15-24 years or ≥25 years) to provide context for the population size estimates. Posterior densities generated from Bayesian nonparametric latent-class models are slightly different each time the models are run. For this reason, the population size estimates and HDIs presented in these 3 tables, as well as Table 5, are rounded to the nearest 100 and the 2 distinct models by age group (ie, 15-24 years and ≥25 years) run for each state will not sum to the overall model representing all ages (ie, ≥15 years).

**Table 2:** State-specific median population size estimates for female sex workers with 80% highest density intervals using Bayesian nonparametric latent-class models, compared with 2018 general population census projections for 7 states in Nigeria.

Age group by state			Median female sex worker PSE^a^, (80% HDI^b^)		General population 2018 census projection^c^ (age ≥15 years)		Median female sex worker PSE/general population, % (80% HDI)
**Akwa Ibom**							
	Total	64,300 (44,100, 84,900)		1,557,841		4.1 (2.8, 5.4)	
	15-24 years	18,200 (11,400, 23,000)		465,126		3.9 (2.5, 4.9)	
	≥25 years	45,200 (32,600, 61,400)		1,092,715		4.1 (3, 5.6)	
**Benue**							
	Total	46,700 (27,500, 113,900)		1,653,910		2.8 (1.7, 6.9)	
	15-24 years	11,000 (9500, 13,400)		624,617		1.8 (1.5, 2.1)	
	≥25years	28,900 (23,500, 35,500)		1,029,293		2.8 (2.3, 3.4)	
**Cross River**							
	Total	15,300 (11,900, 20,000)		1,070,063		1.4 (1.1, 1.9)	
	15-24 years	5500 (4100, 6900)		331,424		1.7 (1.2, 2.1)	
	≥25 years	9600 (7600, 12,200)		738,639		1.3 (1, 1.7)	
**Federal Capital Territory**							
	Total	45,700 (23,100, 56,700)		439,067		10.4 (5.3, 12.9)	
	15-24 years	15,800 (12,200, 21,800)		186,017		8.5 (6.6, 11.7)	
	≥25 years	31,100 (14,700, 38,600)		253,050		12.3 (5.8, 15.3)	
**Lagos**							
	Total	48,200 (30,900, 76,100)		3,858,772		1.2 (0.8, 2)	
	15-24 years	12,100 (7600, 19,600)		955,681		1.3 (0.8, 2.1)	
	≥25 years	32,700 (23,400, 46,800)		2,903,091		1.1 (0.8, 1.6)	
**Nasarawa**							
	Total	55,600 (26,000, 73,700)		569,223		9.8 (4.6, 12.9)	
	15-24 years	22,600 (7100, 29,400)		235,045		9.6 (3, 12.5)	
	≥25 years	42,800 (19,100, 52,000)		334,178		12.8 (5.7, 15.6)	
**Rivers**							
	Total	14,500 (14,100, 15,200)		2,128,841		0.7 (0.7, 0.7)	
	15-24 years	5400 (5200, 5600)		606,665		0.9 (0.9, 0.9)	
	≥25 years	9300 (8900, 10,100)		1,522,176		0.6 (0.6, 0.7)	

^a^PSE: population size estimate.

^b^HDI: highest density interval.

^c^National Population Commission census projections for 2018 population are age- and sex-specific for each.

**Table 3:** State-specific median population size estimates for men who have sex with men with 80% highest density intervals using Bayesian nonparametric latent-class models, compared with 2018 general population census projections for 7 states in Nigeria.

Age group by state		Median men who have sex with men PSE^a^, (80% HDI^b^)	General population 2018 census projection^c^ (age ≥15 years)	Median men who have sex with men PSE/general population, % (80% HDI)
**Akwa Ibom**				
	All	34,600 (12,000, 72,400)	1,594,978	2.2 (0.8, 4.5)
	15-24 years	38,900 (8200, 55,800)	499,067	7.8 (1.6, 11.2)
	≥25 years	17,000 (8900, 31,200)	1,095,911	1.6 (0.8, 2.8)
**Benue**				
	All	10,800 (8000, 13,100)	1,683,863	0.6 (0.5, 0.8)
	15-24 years	2900 (2100, 3600)	650,662	0.4 (0.3, 0.6)
	≥25 years	7500 (5700, 9000)	1,033,201	0.7 (0.6, 0.9)
**Cross River**				
	All	3200 (2700, 3600)	1,046,104	0.3 (0.3, 0.3)
	15-24 years	1400 (1200, 1600)	347,758	0.4 (0.3, 0.5)
	≥25 years	1700 (1500, 1900)	698,346	0.2 (0.2, 0.3)
**Federal Capital Territory**				
	All	8200 (6500, 10,700)	483,100	1.7 (1.3, 2.2)
	15-24 years	3500 (1400, 14,500)	155,809	2.2 (0.9, 9.3)
	≥25 years	6200 (2200, 18,500)	327,291	1.9 (0.7, 5.7)
**Lagos**				
	All	6500 (4900, 8400)	4,746,577	0.1 (0.1, 0.2)
	15-24 years	—^d^	938,061	—^d^
	≥25 years	3800 (2900, 4700)	3,808,516	0.1 (0.1, 0.1)
**Nasarawa**				
	All	5000 (3700, 6400)	477,029	1.0 (0.8, 1.3)
	15-24 years	6500 (2500, 8800)	229,829	2.8 (1.1, 3.8)
	≥25 years	2200 (1900, 2400)	247,200	0.9 (0.8, 1)
**Rivers**				
	All	41,400 (8400, 61,800)	2,354,728	1.8 (0.4, 2.6)
	15-24 years	8000 (2000, 11,300)	649,779	1.2 (0.3, 1.7)
	≥25 years	43,200 (28,300, 63,700)	1,704,949	2.5 (1.7, 3.7)

^a^PSE: population size estimate.

^b^HDI: highest density interval.

^c^National Population Commission census projections for 2018 population are age- and sex-specific for each.

^d^Not available (effective sample size was too small to produce stable, reliable population size estimates).

**Table 4:** State-specific median population size estimates for people who inject drugs with 80% highest density intervals using Bayesian nonparametric latent-class models, compared with 2018 general population census projections for 7 states in Nigeria. People who inject drugs were not disaggregated by sex because the effective sample sizes for women were too small to produce stable, reliable population size estimates.

Age group by state		Median people who inject drugs PSE^a^, (80% HDI^b^)	General population 2018 census projection^c^ (age ≥15 years)	Median people who inject drugs PSE/general population, % (80% HDI)
**Akwa Ibom**				
	All	22,500 (15,100, 30,900)	3,152,819	0.7 (0.5, 1.0)
	15-24 years	5100 (3500, 6000)	964,193	0.5 (0.4, 0.6)
	≥25 years	17,600 (11,800, 23,600)	2,188,626	0.8 (0.5, 1.1)
**Benue**				
	All	27,600 (22,900, 35,600)	3,337,773	0.8 (0.7, 1.1)
	15-24 years	10,200 (7600, 13,900)	1,275,279	0.8 (0.6, 1.1)
	≥25 years	17,900 (14,500, 22,500)	2,062,494	0.9 (0.7, 1.1)
**Cross** **River**				
	All	20,100 (11,500, 25,500)	2,116,167	0.9 (0.5, 1.2)
	15-24 years	6100 (4900, 7500)	679,182	0.9 (0.7, 1.1)
	≥25 years	10,000 (6900, 15,400)	1,436,985	0.7 (0.5, 1.1)
**Federal Capital Territory**				
	All	3400 (2800, 4100)	922,167	0.4 (0.3, 0.4)
	15-24 years	1000 (800, 1300)	341,826	0.3 (0.2, 0.4)
	≥25 years	2200 (1800, 2700)	580,341	0.4 (0.3, 0.5)
**Lagos**				
	All	9400 (7100, 13,400)	8,605,349	0.1 (0.1, 0.2)
	15-24 years	6200 (900, 11,100)	1,893,742	0.3 (0.0, 0.6)
	≥25 years	16,900 (6100, 44,100)	6,711,607	0.3 (0.1, 0.7)
**Nasarawa**				
	All	6900 (5800, 7600)	1,046,252	0.7 (0.6, 0.7)
	15-24 years	1700 (1400, 1800)	464,874	0.4 (0.3, 0.4)
	≥25 years	5200 (4300, 5700)	581,378	0.9 (0.7, 1.0)
**Rivers**				
	All	30,400 (7600, 44,600)	4,483,569	0.7 (0.2, 1.0)
	15-24 years	1700 (400, 2400)	1,256,444	0.1 (0.0, 0.2)
	≥25 years	37,700 (26,200, 50,700)	3,227,125	1.2 (0.8, 1.6)

^a^PSE: population size estimate.

^b^HDI: highest density interval.

^c^National Population Commission census projections for 2018 population are age- and sex-specific for each.

**Table 5:** Comparison of states with 2013 and 2018 population size estimates. The source for the 2013 data is the National Agency for the Control of AIDS [16]. No population size estimates for 2013 were available for Akwa Ibom or Rivers.

Key population by state		2013 PSE^a^, n	Hotspots, n	2018 PSE (80% HDI^b^)	Hotspots, n
**Benue**					
	FSW^c^	10,034	825	46,700 (27,500, 113,900)	1098
	MSM^d^	1018	57	10,800 (8000, 13,100)	265
	PWID^e^	221	32	27,600 (22,900, 35,600)	351
**Cross River**					
	FSW	9858	692	15,300 (11,900, 20,000)	1782
	MSM	276	15	3200 (2700, 3600)	268
	PWID	54	8	20,100 (11,500, 25,500)	616
**Federal Capital Territory**					
	FSW	24,376	1446	45,700 (23,100, 56,700)	977
	MSM	1892	120	8200 (6500, 10,700)	116
	PWID	205	22	3400 (2800, 4100)	111
**Lagos**					
	FSW	46,691	4056	48,200 (30,900, 76,100)	2603
	MSM	2946	191	6500 (4900, 8400)	131
	PWID	1186	95	9400 (7100, 13,400)	240
**Nasarawa**					
	FSW	19,953	1409	55,600 (26,000, 73,700)	990
	MSM	440	19	5000 (3700, 6400)	246
	PWID	414	12	6900 (5800, 7600)	314

^a^PSE: population size estimate.

^b^HDI: highest density interval.

^c^FSW: female sex worker.

^d^MSM: men who have sex with men.

^e^PWID: people who inject drugs.

The modeled estimates were compared with the 2013 population size estimates based on programmatic mapping and enumeration at key population venues, a method that was approved by the GoN [16] (Table 5). Hotspot coverage was broader in our 3S-CRC in 2018 for female sex workers and men who have sex with men in Benue and Cross River, broader for people who inject drugs in all 7 states, and broader in 2013 programmatic mapping for female sex workers and men who have sex with men in Lagos, Nasarawa, and the FCT. In every case, the modeled population size estimates were larger than those from programmatic mapping; only the 2013 population size estimates for female sex workers in Lagos and the FCT were within the 2018 80% HDI.

## Discussion

### Principal Findings

This study represents the first implementation of these sampling and analytic methods to produce large-scale population size estimates for female sex workers, men who have sex with men, and people who inject drugs in Nigeria. Several other applications of 3S-CRC and Bayesian nonparametric latent-class models to estimate female sex worker population sizes in sub-Saharan Africa have been published recently, in South Sudan [20], Kampala, Uganda [21], and Rwanda [22], but to our knowledge no other studies have used these sampling and analytic methods for men who have sex with men or people who inject drugs. Although we were unable to define each hotspot as either urban or rural, and all local government areas thus included urban and rural areas, our study included every local government area in each of the 7 states, and thus represents broad coverage. We demonstrated that these sampling and analytic methods were feasible to implement with appropriate resources and produced reasonable estimates for different key populations in both urban and rural areas. Given the success of our large-scale study with finite resources, these methods could be easily scaled down and applied in smaller settings (eg, cities or provinces).

### Comparison With Prior Work

Of the few population size estimates from Nigeria published before our study, most employed different methods from those presented here. Previous estimates have been generated as a part of programmatic mapping with enumeration at key population venues [15,16]. Several studies used capture-recapture methods for men who have sex with men [16] and male sex workers [19] and were able to report precision, but were limited by geographic focus and probable violation of the independence assumption when only 2 sources were used. Our estimates were generated from 3S-CRC data, and our analysis accounted for dependence in the models, yielding more robust results with precision.

Our population size estimates were larger than previously documented in the 7 PEPFAR priority states in Nigeria. We compared our results with the last population size estimates published by the GoN in 2013 [16]. That study produced population size estimates for 5 of the 7 states in our study. Compared to the 5 states with 2013 estimates that overlap with our 2018 states, our median population size estimates for all men who have sex with men and people who inject drugs in each of the 5 states and female sex workers in 3 of the 5 states were considerably larger than those generated from programmatic mapping and enumeration at key population venues during 2013. In each of the 2 states—FCT and Lagos—where the 2013 female sex worker population size estimates were within the uncertainty bounds of our 2018 study, the number of hotspots contributing to the 2013 estimates was approximately 50% more than the number of hotspots included in our 2018 study. Counts and enumeration from programmatic mapping produce underestimates because only visible individuals who are present at a given venue or hotspot are included, whereas Bayesian statistical models use observed, captured data to estimate the unknown data with uncertainty. In addition, our study had overall broader coverage of hotspots than previous estimates. Extensive hotspot coverage and analysis using Bayesian models that account for heterogeneity in capture probabilities may reflect more accurate population size estimates than previous efforts.

Most of our results fall within expected ranges as a percentage of the general population for each state and key population. We might expect female sex workers in sub-Saharan Africa to comprise 0.4% to 4.3% of the adult female population in urban areas [41]. Our results for 5 of 7 states fall within that range. In the adjacent FCT and Nasarawa areas, where the population size estimates appear to be larger than expected, there is a considerable amount of female sex worker mobility within and between the 2 states, and potential violations of assumptions might provide some possible explanations. For men who have sex with men, Joint United Nations Programme on HIV/AIDS (UNAIDS) 2020 guidance is that the lower bound should be at least 1% of the adult male population [42], although earlier West and Central Africa estimates ranged from 0.05% to 2% in other reports [43]. The overall population size estimates for Akwa Ibom, the FCT, Nasarawa, and Rivers fall within UNAIDS guidance; Benue, Cross River, and Lagos population size estimates are more aligned with the lower bounds of the earlier reported ranges in West and Central African countries. It is possible that the participation of men who have sex with men in our 2018 study might have been impacted by the Same Sex Marriage (Prohibition) Act (SSMPA), signed into law in January 2017 [44]. The SSMPA built on existing laws against sodomy and same-sex marriage and included criminalizing participation in or support of men who have sex with men–friendly organizations and meetings and providing services to men who have sex with men. This might have resulted in fewer hotspots, and fewer men who have sex with men present in those hotspots who were willing to disclose their identity to the study teams composed of their peers. For people who inject drugs, we expected population size estimates to fall within 0.1% to 1.6% of the adult population [8,45], and all of our results met those expectations. Overall, most of our estimates appear reasonable as a percentage of the general population.

We used Bayesian nonparametric latent-class models to analyze the 3S-CRC data. We had several options to analyze our multiple-source capture-recapture data using empirical methods for robust estimates, such as log-linear modeling [24,35,46,47], Bayesian model averaging [48,49], and Bayesian nonparametric latent-class modeling [23,50,51]. Accounting for the heterogeneity of captures and accommodating sparse data are two of the advantages of Bayesian latent-class models over the more traditional log-linear models for analysis of multiple-source capture-recapture data. The Bayesian nonparametric latent-class models account for differences in heterogeneity from capture to capture and combine similar strata into latent classes [23,51]. This feature allows the models to directly estimate the joint distribution, unlike log-linear models that are based on strong assumptions about capture patterns that can result in potentially biased estimates and confidence intervals that display poor coverage. Model selection using the Akaike information criterion or the Bayesian information criterion poses the additional challenge of selecting consistent and correct models [51]. Bayesian model averaging fits all possible log-linear models, weights each model according to the posterior probability, and returns the model-probability–weighted average across many models with uncertainty [48-51]. The Bayesian models are similar in that they inform themselves during Markov chain Monte Carlo sampling, beginning with an infinite number of probability distributions and ending with a smaller, more representative subset of those that fit the data best to produce population size estimates [23,51]. In addition, the Bayesian models can accommodate sparse data (ie, when the total number of individuals with 1 of the 7 patterns of captures across 3 rounds is small or zero). One can chose a method accounting for the independence assumption or the homogeneity assumption, but not both. Bayesian model averaging is best when relaxing independence and the latent-class models are best when relaxing homogeneity. In the context of key populations, individuals have considerably different visibility levels; therefore, we prioritized the homogeneity assumption and opted for the Bayesian nonparametric latent-class models.

### Limitations

The population size estimates presented here are subject to several limitations. First, capture probabilities across rounds might have been heterogeneous. Hotspots, by definition, are key population–friendly locations where members of these groups congregate, so study participants with higher social visibility captured in the first round might have been more likely to be captured again in the second and third rounds. One solution to the issue of capturing only those with strong social visibility might be to have the final capture be part of a respondent-driven sampling survey, with recruitment based on network connections and the ability to reach key population members with poor social visibility (ie, those who do not frequent hotspots). Another option might be to include other types of data sources, such as online social apps, which might have broadened the catchment area, particularly for key population members who do much of their social networking online. Second, unique object (ie, “gift”) acceptance may have influenced our results. A formative assessment with key population communities informed the selection of the unique objects, although it is possible that some objects were less desirable than others and were not accepted by key population members encountered in hotspots, resulting in smaller captures and specious estimates with impractically wide credible sets. Third, compared to other model approaches, Bayesian nonparametric latent-class models are more flexible, which may lead to less stable estimators. Although some may consider this a weakness, we consider it a strength, because there is fundamental uncertainty that is captured by the model in the form of a wider posterior distribution. Finally, although we asked respondents where they resided and whether they traveled to the encounter hotspot or elsewhere for work, the data were insufficient to use for any sort of mobility adjustment to the population size estimates. However, the data did provide some possible explanations for differences among the number of key population members sampled across capture rounds and states. These limitations would likely have resulted in underestimates of population sizes. However, the magnitude of those underestimates might have been mitigated by our broad hotspot coverage throughout the 7 states and stakeholder feedback that many of the key population members who meet sexual or injecting partners online are also found in hotspots. Despite these challenges, our population size estimates were based on 3 high-quality capture rounds analyzed with models that account for capture heterogeneity, and the estimates were endorsed by key stakeholders with local expertise.

### Conclusions

The findings from this study are critical in supporting efforts to respond to the HIV epidemic, as outlined in Nigeria’s National Strategic Framework, as focus shifts from the general population to key populations, suggested by the results of the NAIIS 2018. These population denominator data are essential to align responses and resources from the HIV prevention and treatment programs. In most of West Africa, including Nigeria, most HIV epidemics are not generalized; they are rather mixed epidemics, such that they are focused and propagated within the highest-risk populations yet would be sustained if transmission in either population were interrupted. Indeed, a surge strategy in HIV prevention and treatment is being implemented in each of the 7 PEPFAR priority states. Hotspots identified in this study are also being utilized to map facility-based and community-based programs.

The empirical methods for population size estimates described here provide essential information for planning and implementing targeted HIV prevention, care, and treatment programs. The results of this study demonstrate that this is a method that can be employed in future population size estimate efforts among female sex workers, men who have sex with men, and people who inject drugs in Nigeria and elsewhere. Continuing developments in technology (eg, Shiny apps [50]) that support sampling and analyses of multiple-source capture-recapture by program implementers will increase the accessibility of these methods.

## Abbreviations

3S-CRC: 3-source capture-recapture
CDC: US Centers for Disease Control and Prevention
FCT: Federal Capital Territory
GoN: Government of the Federal Republic of Nigeria
NAIIS: Nigeria HIV/AIDS Indicator and Impact Survey
PEPFAR: President’s Emergency Plan for AIDS Relief
SSMPA: Same Sex Marriage (Prohibition) Act
